# Women’s Perceptions of Living a Traumatic Childbirth Experience and Factors Related to a Birth Experience

**DOI:** 10.3390/ijerph16091654

**Published:** 2019-05-13

**Authors:** Julián Rodríguez-Almagro, Antonio Hernández-Martínez, David Rodríguez-Almagro, José Miguel Quirós-García, Juan Miguel Martínez-Galiano, Juan Gómez-Salgado

**Affiliations:** 1 Department of Nursing, Ciudad Real Nursing Faculty, University of Castilla-La Mancha, 13071 Ciudad Real, Spain; julianj.rodriguez@uclm.es; 2 Department of Obstetrics & Gynaecology, Mancha-Centro Hospital, Alcázar de San Juan, 13600 Ciudad Real, Spain; 3 Department of Emergency, University General Hospital, 13005 Ciudad Real, Spain; davidra81@hotmail.com (D.R.-A.); Josemiguelquiros@hotmail.com (J.M.Q.-G.); 4 Consortium for Biomedical Research in Epidemiology and Public Health (CIBERESP), 28029 Madrid, Spain; jgaliano@ujaen.es; 5 Department of Nursing, University of Jaen, 23071 Jaen, Spain; 6 Department of Sociology, Social Work and Public Health, University of Huelva, 21071 Huelva, Spain; salgado@uhu.es; 7 Safety and Health Postgraduate Programme, Universidad Espíritu Santo, Guayaquil 091650, Ecuador

**Keywords:** childbirth, pregnancy, postpartum, women’s perception of birth experience, maternal mental health, traumatic childbirth, patient safety, quality improvement

## Abstract

Although identified by the World Health Organization (WHO) as a global health priority, maternal mental health does not receive much attention even in the health systems of developed countries. With pregnancy monitoring protocols placing priority on the physical health of the mother, there is a paucity of literature documenting the traumatising effects of the birth process. To address this knowledge gap, this qualitative descriptive study aimed to investigate women perceptions of living a traumatic childbirth experience and the factors related to it. Qualitative data, collected via semi-structured interviews with 32 participants recruited from parent support groups and social media in Spain, were analyzed through a six-phase inductive thematic analysis. Data analysis revealed five major themes―“Birth Plan Compliance”, “Obstetric Problems”, “Mother-Infant Bond”, “Emotional Wounds” and “Perinatal Experiences”—and 13 subthemes. The majority of responses mentioned feelings of being un/misinformed by healthcare personnel, being disrespected and objectified, lack of support, and various problems during childbirth and postpartum. Fear, loneliness, traumatic stress, and depression were recurrent themes in participants’ responses. As the actions of healthcare personnel can substantially impact a birth experience, the study findings strongly suggest the need for proper policies, procedures, training, and support to minimise negative consequences of childbirth.

## 1. Introduction

The majority of approximately 140 million births that occur globally every year are related to women without risk factors for complications either for themselves or their babies at the beginning and throughout the process of labour. Nevertheless, the birth moment is critical to the survival of women and their babies, as the risk of morbidity and mortality can considerably increase when complications arise. In line with the targets of Sustainable Development Goal 3 and the new Global Strategy for Women’s, Children’s and Adolescents’ Health (2016–2030), global health care agencies are expanding their focus to ensure that women and their babies not only survive labour complications should they occur, but also thrive and reach their full potential for health and life [1].

In developed countries, childbirth is a process in which adverse effects are infrequent and unexpected. However, there are instances of unpleasant childbirth experiences that affect maternal and infant morbidity and mortality and that may turn a woman’s experience of childbirth—something expected to be positive—into a traumatic one [2,3]. Some of these situations that occur during childbirth meet the Diagnostic and Statistical Manual for Mental Disorders (DSM-5) criteria for traumatic events [3,4]. Maternal mental health does not receive much attention in today’s health systems, with priority being placed on the physical health of the mother, as can be seen in existing pregnancy monitoring protocols. The World Health Organization [5] identifies maternal mental health as a global health priority. Ten percent of pregnant women and 13% of new mothers experience an undiagnosed mental health disorder [5]. One frequent, yet underdiagnosed, mental health problem with mid and long-term effects on women’s health is post-traumatic stress disorder (PTSD) [6]. PTSD during the perinatal period can occur any time from the beginning of pregnancy to one year after childbirth [7,8].

Various factors make women more likely to develop postpartum PTSD. A recent meta-analysis of risk factors found that subjective negative experiences of childbirth, surgical childbirth and lack of support were associated with PTSD and depression [9]. Another recent systematic review with meta-analysis of PTSD found prevalence rates of 3.3% during pregnancy and an additional 4% during the postpartum period, mainly in relation to traumatic events during childbirth [10].

A traumatic birth is considered an event involving injuries, and even serious danger to or the death of the mother or child. Indirect exposure to trauma has been associated with a series of adverse psychological responses, including PTSD, which is defined by the American Psychological Association (APA) in the Diagnostic and Statistical Manual for Mental Disorders (DSM-5) [3,11,12]. Feelings of distress, unwanted memories, fear, guilt and even shame are characteristic of traumatic events during the perinatal experience [3,11,12].

Childbirth is a critical role transition in women’s lives. Women undergo physical, psychological and sociocultural changes during pregnancy, childbirth and the postpartum that can affect their own health and wellbeing as well as the baby’s health and wellbeing [13].

The World Health Organization (WHO) recognises a “positive childbirth experience” as a significant end point for all women undergoing labour. It defines a positive childbirth experience as one that fulfils or exceeds a woman’s prior personal and sociocultural beliefs and expectations, including giving birth to a healthy baby in a clinically and psychologically safe environment with continuity of practical and emotional support from a birth companion(s) and kind, technically competent clinical staff. It is based on the premise that most women want a physiological labour and birth, and to have a sense of personal achievement and control through involvement in decision-making, even when medical interventions are needed or wanted [14].

This comprehensive view on essential intrapartum care brings together new and existing World Health Organization (WHO) recommendations that, when delivered as a package, will ensure good-quality and evidence-based care irrespective of the setting or level of health care. The recommendations presented are neither country nor region specific and acknowledge the variations that exist globally as to the level of available health services within and between countries. The WHO highlights the importance of woman-centred care to optimise the experience of labour and childbirth for women and their babies through a holistic, human rights-based approach. It introduces a global model of intrapartum care, which takes into account the complexity and diverse nature of prevailing models of care and contemporary practice [14].

Therefore, and as a result of the above, we consider it necessary to investigate women’s perceptions of living a traumatic childbirth experience and the factors related to a birth experience, since these types of studies are very novel and can help to modify the health policies of the different agencies and of all the personnel who work in them and who, in one way or another, are at the bedside of the women who are about to give birth.

## 2. Material and Methods

To collect information, consecutive sampling was used, and an appeal was launched through the main women’s associations and the Spanish Federation of Midwives Associations (Federación de Asociaciones de Matrones de España, or FAME) as well as its member associations, which involved midwives in disseminating the project and attracting participants. Once the study subjects had been selected and had agreed to take part, they were provided instructions to fill in the informed consent form if they wished to take part in the study. Women were eligible to take part if they were aged 18 or over. 32 women agreed to take part, and all of them were interviewed.

We carried out a qualitative descriptive study [15] based on thematic analysis [16]. Semi-structured interviews lasting approximately 45 min were conducted with 32 women. They were invited to talk about the birth of their baby and how it had subsequently affected their lives by responding in their own words to the main question “describe your experience of the birth and what you found traumatising”. The interview questions (Annex 1) were designed by the research team based on their clinical experience as midwives, including perceptions, thoughts and feelings developed during this period. This study was approved by the Ethical Committee on Clinical Research (CEIC, for its Spanish acronym) of the Mancha-Centro Centre, with ethical code 69-C.

**Annex 1.** Semi-structured interview questions.1. How was your childbirth experience?2. How did you feel during childbirth and postpartum?3. Have these feelings changed over time?4. How do you feel now?5. If there have been any changes, what helped you? What held you back?6. Apart from this interview, have you had any other opportunities to express your feelings on the entire childbirth process and the postpartum period?7. Can you suggest anything else you think would be useful for another woman in your situation?

Audio recordings were made and field notes were taken; recordings were subsequently transcribed [17]. In order to maintain their confidentiality, all of the women were assigned a pseudonym consisting of the letter W for woman and a number from 1 to 32, corresponding to the total number of women that participated in the study. The women were informed, by means of the patient information sheet, that assistance from healthcare professionals was available if they felt distressed and the interviewer reminded them that they could stop the interview at any time if they did not wish to answer questions. The interviewer was not known to the participants prior to the interview.

Women’s descriptions of trauma were analysed using a six-phase inductive thematic analysis process described by Braun and Clarke [16]. Phase one involved becoming familiar with the data by reading and re-reading and noting initial ideas. In phase two, initial codes were generated and data relevant to each code were collated. Phase three of the process involved collating the codes into potential themes. These themes were reviewed in phase four to ensure they were consistent in the coded extracts and across the entire data set. In phase five, themes were defined and named using words and phrases. Phase six involved selecting extract examples to illustrate the themes and relating the analysis to the research question and the literature. Three researchers participated in the thematic analysis process to ensure consistency in analysis and findings [16,18].

During the process, the criteria for methodological rigour of credibility, dependability, confirmability and transferability were observed [19]. All authors participated in the validation of the results, questioning each step of the analysis to check any alternative interpretations. The analysis was discussed until agreement was reached.

## 3. Results

A total 32 women with a mean age of 35.5 years (SD: 4.79, range 18–43) participated in the interviews.

Five major themes were identified in the data: “Birth Plan Compliance”, “Obstetric Problems”, “Mother-Infant Bond”, “Emotional Wounds” and “Perinatal Experiences”. Within these 5 main themes, 13 subthemes were identified (Table 1). The content of each major theme and subtheme are described below using quotes from participants.

### 3.1. Birth Plan Compliance

Compliance with the birth plan was a constant theme in the women’s answers. Women need to know who is treating them, and above all need to know that they are not being misled with regard to the initial birth plan. Women stressed the importance of the issue regarding when manoeuvres and techniques such as membrane sweeps, fundal pressure, episiotomies and use of forceps and spatulas are carried out without their consent and without receiving any explanation of the manoeuvre or the medical reason for it.

W4 “I suspect that the gynaecologist did a membrane sweep without my consent and without telling me. It was very painful. During the contractions I couldn’t help but cry out, and they basically treated me like an animal, shouting at me and telling me to be quiet, saying that if everyone cried out like I did they would go deaf. Then they did an epidural and a rupture of membranes without my consent and without telling me what they were doing. Finally, in the second stage of labour, after having said I didn’t want an episiotomy, the gynaecologist said there was foetal distress and that I would have to a forceps delivery and of course an episiotomy, which was very big. I have to say that the whole process from going into labour to giving birth only took seven hours…”.

W18 “The birth was not respected at all, I was misled starting from the membrane sweep, which was not explained at all, I was mistreated because it was a public holiday, they didn’t let anybody in, they applied fundal pressure, the way they treated me was unacceptable, they didn’t let me push, they cut me very early, I didn’t participate in anything, they took my baby out using a ventouse because they were in a rush, they didn’t let me see him, and when they did they told me to stop crying about it. They removed the placenta manually, I fainted due to being in labour for 24 h, I wasn’t allowed to see the baby for another 2 h.”.

W6 “I arrived at 8 cm and was managing to deal with the pain. My idea was to have a natural birth with no epidural. The gynaecologist and nurse held me down without my consent to examine me. I felt a lot of pain and fear. From then on, the contractions changed, the baby wasn’t coming. I asked for an epidural because I could no longer control the pain because I was afraid of the doctors. They weren’t respecting what was supposed to be my moment. There was lateral deviation of the epidural, back pain, constant painful contractions. The C-section was not respected, and I was separated from the baby once it was born. Also, the father wasn’t allowed to be present at the C-section.”.

W32 “The gynaecologist ruptured my membranes without my permission, ignoring my requests and my agreement with the midwife. This caused a conflict between the two (midwife and gynaecologist). At no time did they give me any medical justification.”.

W23 “My birth was not respected at all. The midwife told me that I didn’t know how to push, and it was my fault that the baby wasn’t coming out. He tried to apply fundal pressure without explaining what he was doing. When I refused, he ignored me and tried to do it again. When I refused for the second time, he lost interest in me. It was the most brutal thing I have ever experienced.”.

The solution in these cases is simple: Giving a simple explanation of each technique at each stage, as well as the medical reason for it, could make women feel safer, respected, and not like they were being deceived, which could make them more collaborative in their birth process.

Within the birth plan compliance theme, the subtheme *Healthcare staff not empathetic* was the most frequently commented on and was mentioned by nearly all participants. In the hospital, the need to know what happened and what was currently happening was pressing: Women need to know who is treating them at all times.

For example, “it was horrible because I didn’t feel respected by the healthcare personnel that treated me, they came in and touched me and didn’t even introduce themselves” (W2).

W5 told us that there were “a lot of strangers in the operating room, and my partner wasn’t there. I didn’t know who was who, the gynaecologist, the front desk personnel, the anaesthetist…”.

W17 “During the dilation, the whole thing seemed like a showroom for groups of students, and I wasn’t asked if I was OK with it. So it was not a great experience”.

W15 “I would like the care given to women giving birth in hospitals to be improved, they are treated like cattle. There’s no empathy of any kind”.

W9 “It was a frustrating experience. They didn’t respect my birth plan. There were loads of people in the delivery room, going in and out all the time. Nobody introduced themselves or explained what they were doing”.

In cases like this, the solution is to always introduce oneself, look the patient in the eye and explain everything that is going to happen and all procedures that are going to be performed on them.

Another subtheme within birth plan compliance is *feelings of being misinformed*, women desired information at all phases of their experience. Women wanted to know what, how, and why everything happened. Communication was a common theme in the interviews, not only with regard to what information should be communicated, but also when and how it should be communicated.

In their answers, we saw that women arrive at hospital with predefined notions of what their ideal birth is going to be like, and this is often discussed with healthcare personnel. So, what are we doing wrong? Why are they not listened to? Let us look at some of their answers:

W19: “My pregnancy was amazing, but my daughter was in a breech position. I asked the hospital if I could still give birth naturally and they told me that it would be no problem. But it was all a lie, because when I arrived at hospital to give birth, they only gave me 7 h to dilate before taking me for a C-section for no reason, because we were both in perfect condition. Throughout the dilation period I had to put up with comments from midwives, some gynaecologists and other personnel criticising and judging my decision to give birth to my daughter naturally.”

W20: “Normal vaginal birth, not very respected. Unfortunately, my birth experience was like a lot of births: unnecessary procedures and having to negotiate every little thing rather than focusing on my baby and giving birth. In the end, the pressure from the hospital, an unpleasant, unfamiliar environment and the fact my times weren’t respected (when I was examined upon arrival, the midwife ruptured my membranes without my consent) led to me getting an epidural, which complicated things and because of that, as well as the fact that they didn’t want to “waste time”, my daughter was born using a ventouse.”

Are all of these procedures really necessary, and if so, why are they done without any explanations? We need to respect women and their predefined birth plans, and we need to try and explain any unfamiliar procedures if the woman is not aware of them. These explanations will help women understand that sometimes, the birth plan can change, which is not a problem as long as they are properly informed. This will help women accept the birth plan as we will act with their implicit consent.

### 3.2. Obstetric Problems

Another main theme that came up when analysing the answers were obstetric problems. Within this, there are two subthemes: Problems during childbirth and problems postpartum.

The subtheme *problems during childbirth* contains problems stemming from episiotomies, lacerations, pain due to pain relief not working or not waiting for it to work, or being given an epidural at the wrong time or with improper technique.

W14: “I had a C-section and they began cutting before the anaesthesia had taken effect (I told them that I could feel the wet cloth they rubbed over my belly, but they still began cutting) so I felt the pain of them cutting me and I could feel their hands opening my belly.”

W27: “It was horrible, I tear up when I think back to it. The pain was awful, and they had to administer the epidural more than four times because my doctor had difficulty doing it.”

W3: “Giving birth was the worst day of my life. The midwife was very caring, but the trainee was anything but. The plan was not complied with. The person that sewed me up (episiotomy) was very rude, he told me to be quiet and complained that women always say that he doesn’t sew them up properly.”

W17: “It was a natural vaginal birth with no analgesia. It was very quick but very, very painful, the pain from the episiotomy was horrible, and even though they gave me a local anaesthetic for the stitches, it hurt.”

The solution to these types of problems is simple and lies in waiting the correct amount of time for the anaesthesia to take full effect. In the case of births attended by staff undergoing training, anaesthesia must always be administered under the supervision of the professionals responsible for the woman’s care in order to correct technical aspects as well as the care provided to the patient.

When analysing the subtheme *problems postpartum* we found problems related to the pelvic floor, such as constipation, incontinence and dyspareunia, as well as problems with sex.

W11: “Since my first pregnancy I have had a lot of problems with haemorrhoids and urine infections. Until I went to a private specialist to prepare for my second childbirth, I didn’t realise that the cause of these problems was that my pelvic floor hadn’t recovered.”

W27: “For the second pregnancy I had a lot of problems with incontinence, I think my pelvic floor was more damaged by the second pregnancy... I think more importance needs to be placed on this.”

W31: “Since giving birth I have had a lot of vaginal dryness which affects my sex life, as well as problems with incontinence, especially when I walk a lot or during physical activity.”.

### 3.3. Mother–Infant Bond

Some women reported a special bond between the mother and the newborn. This bond can be promoted through skin-to-skin contact and breastfeeding, which form the two subthemes within this theme. The women told us that this theme is special because it has a positive influence on the psychological relationship between mother and baby and they ask for respect in order to strengthen this bond as much possible and not break it, as occurs in certain hospitals.

In the *skin-to-skin* subtheme, women expressed a particular need to make skin-to-skin contact with their baby:

W5: “The C-section was very unpleasant; they didn’t let me touch the baby. It’s a shame because in some hospitals I have seen that they let mothers have skin-to-skin contact after the C-section, something which helps the baby and the mother psychologically.”

W8: “Giving birth seemed like a cold experience. I was alone in the operating room with my hands tied, I was allowed to see and kiss the baby for two minutes then they took her away with my husband and because of nerves or whatever he didn’t remember to make skin-to-skin contact and the nurse didn’t remind him.”

W22: “It was a bad experience because they separated me from my babies when they were born and even though they kept saying that skin-to-skin was so important, they took my daughters away and put them in an incubator without even showing me them so I could see they were okay. I felt alone, without my daughters or my husband, who they didn’t let in. I had to keep my own spirits up otherwise I would have been really upset.”

W29: “I wasn’t able to make skin-to-skin contact, I didn’t see my baby come out and didn’t meet him until three hours later. I couldn’t be with my husband during the C-section or in the hours following the procedure. My son’s temperature went down and they had to take him to an incubator (accompanied by his father); by the time I saw him he was already in the cot, dressed and covered up. I think that if I had been allowed to do skin-to-skin, even in the recovery room, his temperature wouldn’t have gone down. We didn’t get that initial contact.”.

Another important subtheme for women is *breastfeeding*. All of them wanted to breastfeed their babies and some of them felt their efforts were not supported. They also mentioned that breastfeeding should begin as early as possible. Immediately beginning to breastfeed helps to strengthen the mother–infant bond. The women told us that are a lot of obstacles when it comes to starting to breastfeed, that it should be a priority in hospitals, and that artificial feeding should not be defended like it is in some hospitals.

We must place emphasis not only getting a good latch, but also on giving correct advice from the start, problems with tongue-tie are common and can be corrected if they are noticed.

The women also stressed that the benefits of breastfeeding over artificial feeding are widely known, so another priority for hospitals should be to correct their guidelines and look for new ones that help reinforce breastfeeding over artificial feeding.

W14: “Breastfeeding was very, very hard. Extreme tiredness. I felt powerless. Sometimes I wanted to disappear. External pressure to begin artificial feeding and you start to feel unsure of yourself and wonder whether you’re doing it right by breastfeeding every 15 or 20 min. I wondered if they were really hungry and if I was being stubborn with breastfeeding. In the end I managed to stick with breastfeeding. They’re now 14 months old and they continue to feed on demand (I’m still criticised now).”

W19: “I would just point out the problems I had with breastfeeding at the start (since I later found out that the baby had tongue-tie, hence the pain, cracks, blisters, etc.) and the lack of support: Despite looking everywhere to find an advisor who could deal with the problem, I am still doing mixed feeding because my baby is too used to the supplement (he is five months), which often gets me down and limits me, as sometimes he doesn’t want to breastfeed and feeding becomes a nightmare. I like to think that maybe if I had been given the correct advice from the start (the midwives at the hospital would just say that he was latching on correctly, they never looked at his tongue), my breastfeeding would have been a lot different.”

W17: “In the first few weeks the baby didn’t breastfeed enough and, in the end, due to external pressures, I had to supplement with formula, something which I had a bad time with. Finally, against everyone’s advice (the father, the grandmother, etc.), I managed to completely eliminate formula, and continued to breastfeed until my baby turned two. I think that there is not enough information about breastfeeding and mums aren’t prepared for the problems that can come up.”.

### 3.4. Emotional Wounds

Another important theme was emotional wounds. Women told us that there is an emotional impact that occurs during childbirth and postpartum, which they reported as emotional problems stemming from the entire process. These emotional problems vary from general feelings like crying all the time about anything to fear surrounding problems that could come up. Several of those interviewed suggest solutions like pregnancy and breastfeeding support groups, whether before or after giving birth. They also mention that support from family was good in this regard as it helps them through times when they feel more emotional.

W6: “Postpartum was more difficult for me, more down to emotional problems than physical ones, I had no idea how much having a child would change my life, hours and hours of breastfeeding, holding, colic... I don’t think we were told about all of that in antenatal classes and I think it is important. For me, the breastfeeding support group at the health centre helped a lot.”

W3: “There were times I cried because I felt like the situation was too much for me physically and because of caring for the baby, but thanks to my husband and my family I began to feel better.”

W24: “The fact I was not able to give birth naturally affected me emotionally. I think I will only get over it if I get pregnant again and manage to have a natural birth.”

W23: “Postpartum brings back very bad memories, I was very happy to be with my baby but felt sadness, wanting to do everything but not being able to, visits were just a nuisance when you just want to be alone with your partner and baby to start breastfeeding, getting obsolete advice.”

W32: “I was so scared about anything going wrong that I didn’t enjoy my pregnancy. I had a long labour (induced). I was sad and cried a lot. I couldn’t breastfeed and felt very guilty.”

This main theme Emotional wounds includes 4 subthemes: *Fear of things not going well, Stress and frustration, Loneliness, and Depression.* We will now take a look at teach one.

Within the subtheme *Fear of things not going well*, most women described feelings of uncertainty about what to do and what not to do with the baby. The participants told us that there is constant worry which, in some cases, stems from prior experiences such as miscarriages or bad birth experiences, as well as fear about the epidural, the baby’s development and fear of the unknown. These situations that women experience must be taken into account, and we must focus on giving more information on the whole process.

W2: “I was scared of the epidural, of the baby not gaining weight, not knowing how to carry him, and I am still scared about a vaginal birth, because I haven’t had one due to fear about the pain or the baby not coming out, and I was scared throughout the pregnancy about something going wrong because I had a miscarriage before becoming pregnant.”

W8 “It was very hard psychologically because of fears about caring for the baby and not knowing if you’re doing it properly, and the lack of rest.”

W20 “Sometimes the situation overwhelms you and you just cry about everything and feel very small in front of this small person who is going to be your main concern for the rest of your life, the whole thing is scary.”

The women interviewed also told us that they felt *stress and frustration*, which we will treat as a subtheme of the main theme Emotional wounds. They reported feelings of uncertainty and exhaustion which make them feel stressed, due on the one hand to not being able to resolve situations they are faced with, and on the other, frustration stemming from dissatisfaction at not being able to live out the idealised situations with their baby they had imagined prior to giving birth.

W4: “Mothers aren’t given much consideration in hospitals. For us, it’s a brutal experience and in some places human warmth is really lacking.”

W8: “Emotionally, I was crushed, inconsolable: I had no confidence.”

W22: “Overall, I had a bad experience and just thinking about becoming pregnant again makes me panic and stress when it is supposed to be something beautiful. I remember the two days that everything took with sadness, frustration, fear and a lot of pain.”

Women express feelings of *loneliness* as a constant throughout the process of labour and postpartum, and we have classed this as another subtheme within Emotional wounds.

The participants told us they felt especially alone postpartum and that they did not get enough support in the first few months. Even those that do can feel like they are not enough and feel disappointed on an emotional level with their partners. We should therefore make a point of giving training not only to mothers but also to their partners and close family so that women do not feel alone and so that they get the support they need.

W27: “I am finding the postpartum period hard, especially since the dad went back to work. Accepting that my daughter depends on me and everything else is secondary is hard.”

W4: “The early days of being a mum were very hard, I had lots of doubts and above all, I felt very lonely despite being surrounded by my family.”

W3: “I felt like I wasn’t enough, and I felt lonely, despite having the support of my partner and the midwives... I felt sad, apathetic.”

W18: “Lonely and disappointed with my partner, no emotional support, or any support of any kind, but especially emotional.”

The final subtheme under Emotional wounds, and perhaps the most important consequence stemming from all of the foregoing, is *depression*. Women’s reports of depression were a consistent theme and attention must be paid to this, as depression has high rates of morbidity and mortality. The participants themselves give us the solution here: The fact that it seems like everything is going to be wonderful after the birth means that this ends up not being the case and the new mother thinks that everything is going badly. Part of the preparations for childbirth should focus on the postpartum period and telling them that not everything will be easy, as a lot of women suffer with this, and it can end up having an impact on their relationship with their partner or even breaking up the relationship.

W9: “My postpartum was very sad, I would cry all day, saying sorry to my baby for not being brave and for not speaking up and making him go through all of that. I think I had postpartum depression, but I didn’t go to the doctor for a diagnosis.”.

W27: “The hardest thing postpartum was the pressure from people around me, everyone knows everything and judges and although I thought I could handle anything, in the end I couldn’t because I became very withdrawn due to exhaustion and the negative comments about how I was caring for the babies. I had postpartum depression and had to be on medication for more than a year.”

W13: “My last pregnancy was somewhat different to my first because this time I didn’t have the support of the father, I was very depressed and felt sad and lonely, giving birth was very hard because it was very painful.”

W2: “Breastfeeding starting off badly and I am still having difficulties, though we’re still doing it at night. I had an episode of postpartum depression with delirium and I’m still getting psychological treatment.”

W5: “After a risky pregnancy and having a premature baby, I couldn’t breastfeed my baby, which made me feel depressed. I had postpartum depression which I still have today, because my husband also got depressed and now, we don’t have a good relationship.”

W9: “After giving birth I became totally depressed, I didn’t want anyone to see me or visit me, I didn’t want to go out, I cried a lot, I didn’t feel happy about having the baby even though I couldn’t wait for the baby to be born. At the same time, I couldn’t forgive myself, I felt disappointed in myself, I couldn’t enjoy myself after wanting a child for more than 13 years, and when my baby finally came I didn’t know what was wrong with me, I was depressed for almost 2 and a half years.”

W29: “I didn’t receive any psychological treatment or support... taking into account my age (23)... I now understand the deep depression I fell into, which lasted two years and separated me from my partner because all I saw were responsibilities. The change is too drastic.”.

### 3.5. Perinatal Experiences

The final main theme is called Perinatal experiences and includes the following subthemes: Traumatic experience, obstetric violence and finally wonderful experience.

*Traumatic experience* refers to women’s description of the perinatal process as traumatic and something which has stayed with them. On several occasions, the women themselves use the term ‘traumatic’ in their descriptions, often in relation to C-sections or instrumental births, the use of techniques such as applying fundal pressure, episiotomies, and so on. The solution could involve giving more information on the entire process and all of the unforeseen events that can come up. This will help create more realistic expectations of childbirth, rather than an idealised view.

W23: “Traumatic. Vomiting, nausea, absolute rest, admitted to hospital due to contractions. Both were premature births, I had placental abruption with the first and with the second I had general anaesthesia to get the baby out with a previous caesarean and vertical incision.”

W27: “A traumatic experience. My first childbirth was a disaster: induction, my partner wasn’t allowed in, the epidural didn’t work, fundal pressure was applied, I had an episiotomy, no skin-to-skin contact.”

W31: “My son was born with congenital torticollis and two injuries to his head and cheek from the spatulas used by the gynaecologist. I had an episiotomy, but the spatulas tore the entire perineum and anal area as well as part of my thigh. If it happened to me now, I would report the gynaecologist, but at the time all I wanted to do was cry.”

W18: “Normal pregnancies but very traumatic childbirths. The first because I was trying to give birth naturally (but medicated) for almost a whole day; eventually I needed an emergency C-section due to foetal distress and the baby ended up in intensive care for 10 days. The second childbirth was premature at week 31 + 6 due to a premature rupture of membranes. The baby was in intensive care for a month and got enterocolitis while admitted.”

W27: “My childbirths were quite traumatic because I wanted them to be natural and they didn’t fit in the birth canal. After many hours and being fully dilated they decided on a Caesarean. I feel like they robbed me of my childbirths.”

In the perinatal experiences theme and going one level beyond a traumatic experience, some women reported having experienced *Obstetric violence*, or feeling violated. This sensation comes from multiple vaginal examinations by multiple professionals during the childbirth process. In this regard, we need to assess whether certain examinations are really necessary and reduce them to a minimum. Where healthcare professionals are undergoing training, women must give their consent and always be informed which professional is attending to them.

W12: “When I got to the hospital, they saw on the monitors that I was having lots of contractions but wasn’t dilating, so the midwife did a procedure to begin dilating at each examination, and there were a lot of them, I lost count. I felt violated over and over again.”

W16: “It was a no-epidural birth but there was a lot of intervention, even though I was fully dilated when I arrived at hospital. I was given oxytocin, fundal pressure was applied, and they had their hands in me the whole time. I experienced a lot of pain and I was emotionally affected too, although everything went well.”

W25: “all this time they did endless vaginal examinations, another thing I asked them not to do unless really necessary. I felt violated and humiliated.”

W28: “the gynaecologist was barbaric, I don’t think a woman that has just given birth should be examined like that, inserting a hand that roughly, I felt violated.”

W10: “a lot of students and doctors came to examine me, it was very uncomfortable and happened during the early morning, morning and night shifts. I saw at least 15 people and they all examined me, it was very uncomfortable, I felt like I was being continually violated.”

W30: “I suffered obstetric violence, with multiple very painful examinations with absolutely no empathy throughout the dilation process.”

W1: “As soon as I got to hospital after my waters broke, about five people touched me without looking me in the eye, they didn’t even introduce themselves, I felt violated.”

The final subtheme includes women who rate their perinatal process as a *wonderful experience*. This type of childbirth is usually associated with a quick, pain-free, instrument-free birth, normally accompanied by their partner. Women that rated their childbirth as wonderful were those that received excellent care and empathy from healthcare personnel.

W7: “My experience was wonderful both times I gave birth. They were natural births with no epidural, they were quite quick, and both mother and baby’s needs were respected. The first time, the gynaecologist that I saw throughout my pregnancy was there, as well as a midwife and the father. The second time, there were two midwives, and the father was present, the gynaecologist wasn’t there due to health problems. Everything went wonderfully, both babies were born healthy and I recovered very quickly.”

W13: “During labour I felt very protected by all of the staff. It was a magnificent experience. The maternal education given at the end of pregnancy should start with a session explaining what is going to happen and what is normal.”

W21: “The birth of my daughter was beautiful and very quick; it was a completely natural birth.”

W26: “The whole process from being admitted to holding my baby in my arms took three and a half hours. It was an excellent birth, with excellent care. For me, it was a great experience. I was respected and cared for.”.

## 4. Discussion

This study explores women’s experiences of traumatic childbirth and the factors related with the experience of childbirth. Our findings fill a gap in the literature about these women’s perceptions of care and about recommendations to form the basis of care based on their experiences.

Some of the five themes and 13 subthemes identified in our study are consistent with the findings of existing qualitative studies [20,21,22]. Research with women who had traumatic or complicated births, or who had a negative experience of childbirth, found similar themes: fear, pain, a need for information, compassion, care, sensitivity, etc. [20,21,22].

Our results are very much in line with Ayers et al. [23], whose study features women with symptoms described as traumatic, their bodies treated as a simple object that anybody can touch, undergoing multiple vaginal examinations throughout the birth process, and feeling extremely stressed from the physical pain and sometimes even depressed.

Negative emotions are frequently mentioned in qualitative studies. Some examples of negative emotions experienced during childbirth and postpartum are feeling powerless, feelings of humiliation and shock, as well as feeling violated and completely dehumanised [7,24,25], as we have described in this study.

The majority of women in our study reported pain of varying intensities during labour and their expectations of the process were confounded, making the experience more traumatic. The experience of childbirth may have a significant impact on women’s mental health, especially if they experienced pain or the birth did not meet their expectations due to complications, as described by Soet et al. [26].

The women’s comments reveal that they were not prepared for complications and received inadequate care during childbirth. The feeling of not being seen or listened to during childbirth contributed to a negative experience, as occurs in the study done by Henriksen et al. [27].

In short, the results suggest that many parturients in this study felt they have been crassly objectified and deprived of their human dignity as individuals. The findings suggest that there was no informed consent of the parturient to allow individuals who were not part of the direct care team (e.g., students, non-healthcare personnel) to be in the labour room. While the participants did not say whether the direct care team had or did not have identification nametags, they complained that they failed to respectfully introduce themselves to the parturient women, thereby increasing the parturients’ level of confusion and anxiety. It appears that the care personnel failed to explain every procedure and the intent to medicate the parturient, as well as the risks and benefits of the different procedures and medications. In the absence of such information, a parturient cannot make an informed decision to accept or refuse an intervention. Finally, the results suggest that the health care team failed to obtain from parturients an informed consent for procedures.

## 5. Conclusions

### Limitations and Future Directions

The study participants were recruited from specific parent support groups and through social media in Spain; this sampling does not allow pinpointing a geographic area for future policy recommendations. While not generalisable due to the qualitative nature of the study, the findings point out the need for a follow-up with a large-scale population survey to assess the actual magnitude of the traumatic birth experience in a particular region of Spain, before moving to a national level study.

We intend to follow up with a large scale survey in the province of Ciudad Real, part of the autonomous community of Castile-La Mancha, Spain, with the aim to assess to what extent the procedural deficiencies identified and described in this study (e.g., no self-introduction of the care team to the parturient, no explanation of the risks and benefits of procedures and medications, no informed consent for procedures and medications, no informed consent for non-direct health care personnel to be in the labour room) are in fact present in the birthing practice at the clinics of Ciudad Real.

If future findings confirm a significant presence of traumatic birth experiences due to the above-mentioned deficiencies, we will recommend and advocate for policy changes. Recommendations would include increasing and improving the information given in perinatal health classes, focusing on the entire perinatal process and including the various complications that can occur during labour, as well as increasing training for all personnel caring for the parturients (gynaecologists, midwives, nurses) in relation to breastfeeding and its benefits. However beneficial, mere recommendations might not change the status quo.

Mandating and enforcing procedural policies that respect the dignity of parturients could, in fact, significantly reduce the traumatic birth experience. Perhaps mandating such knowledge and skills in the licensing process and the continuing education of the medical and nursing personnel would be a good starting point.

## Figures and Tables

**Table 1:** Themes and subthemes.

Themes	Subthemes			
Birth Plan Compliance	Healthcare staff not empathetic	Feelings of being misinformed		
Obstetric Problems	Childbirth	Postpartum		
Mother-Infant Bond	Skin-to-skin	Breastfeeding		
Emotional Wounds	Fear of things not going well	Loneliness	Stress and frustration	Depression
Perinatal Experiences	Traumatic	Obstetric Violation	Wonderful	
